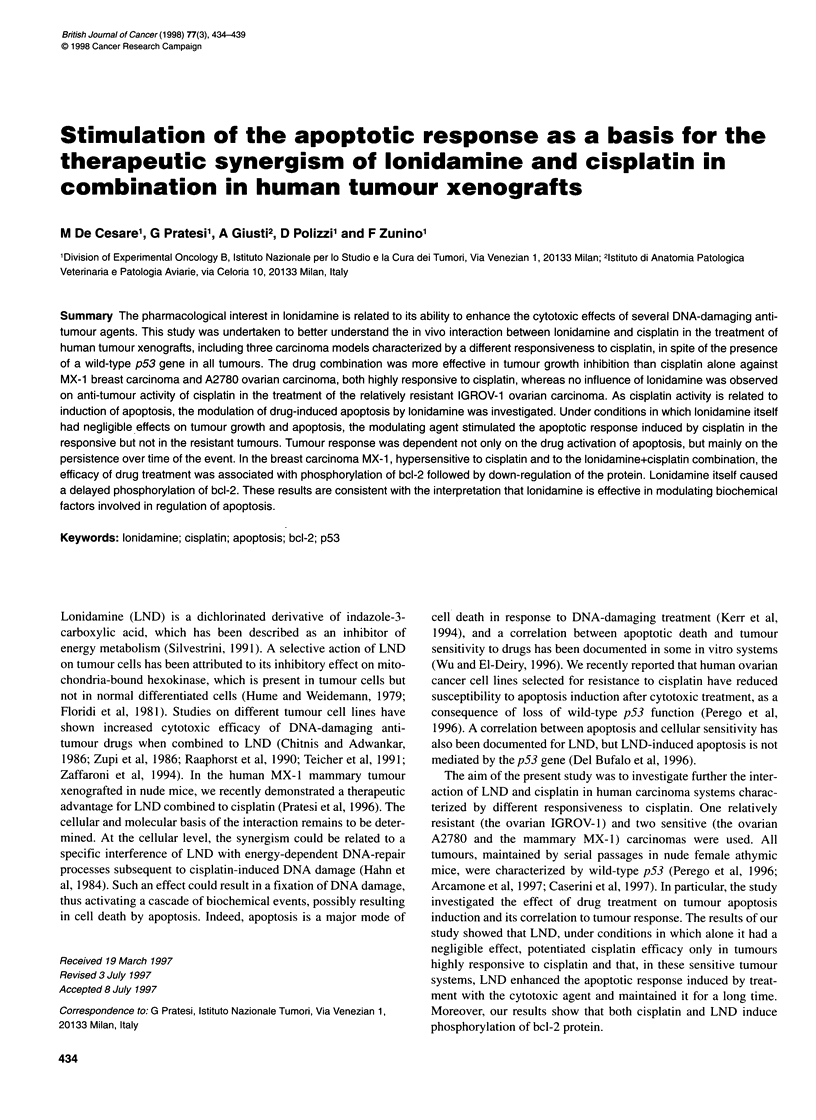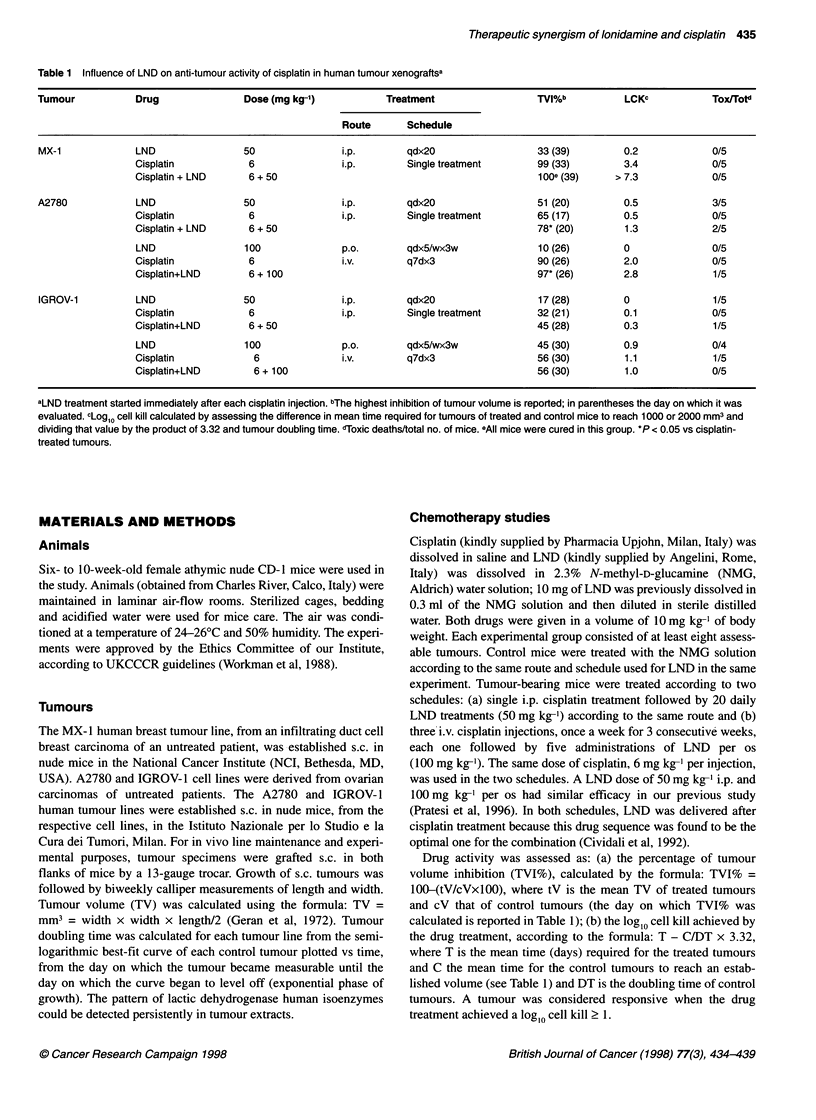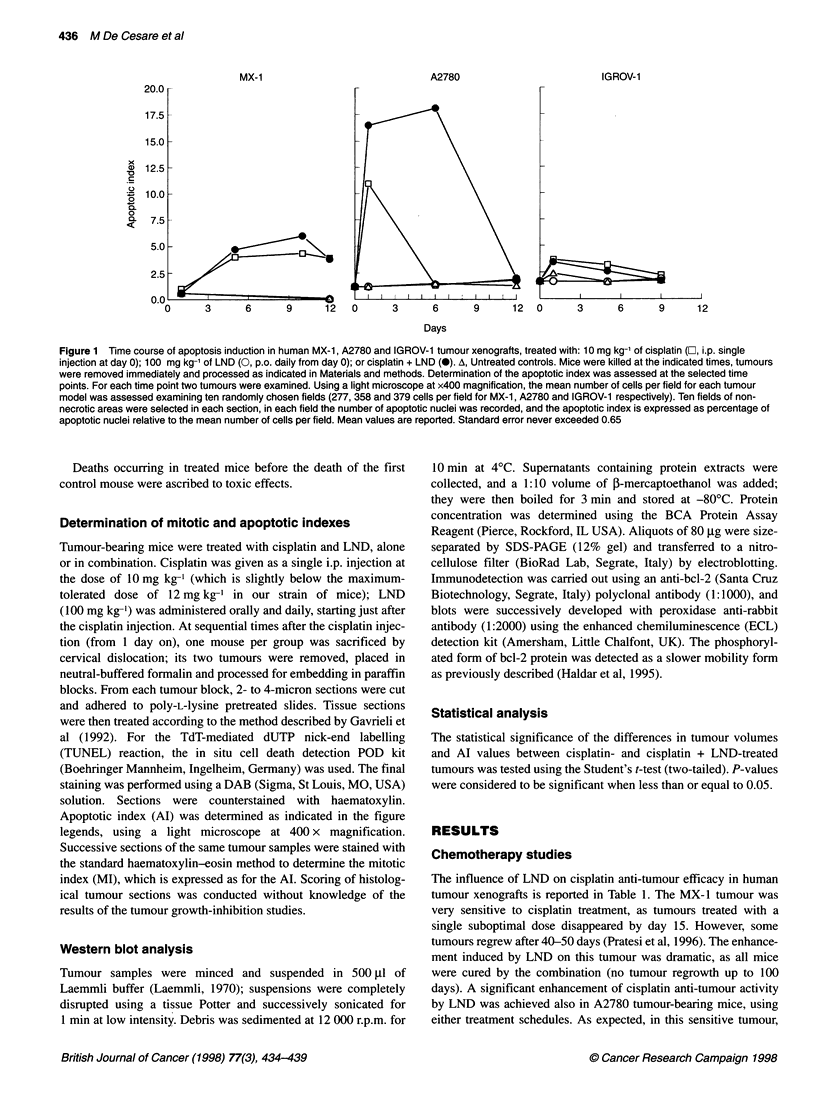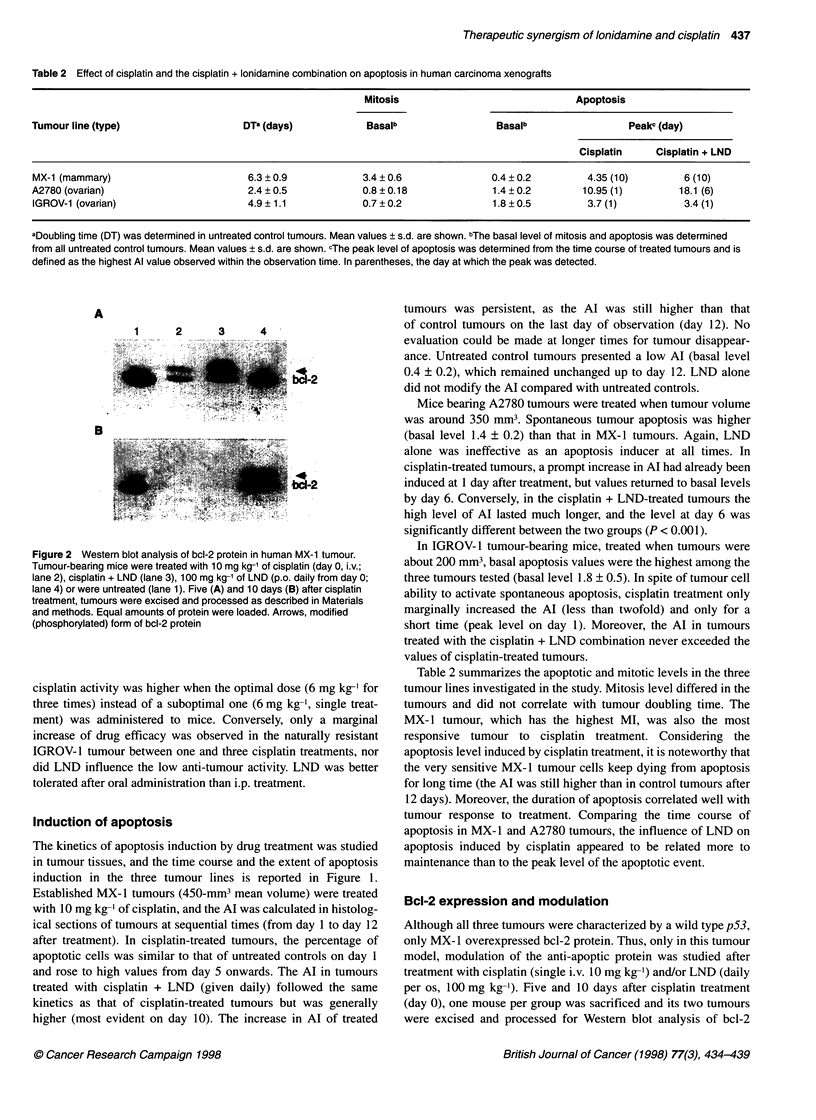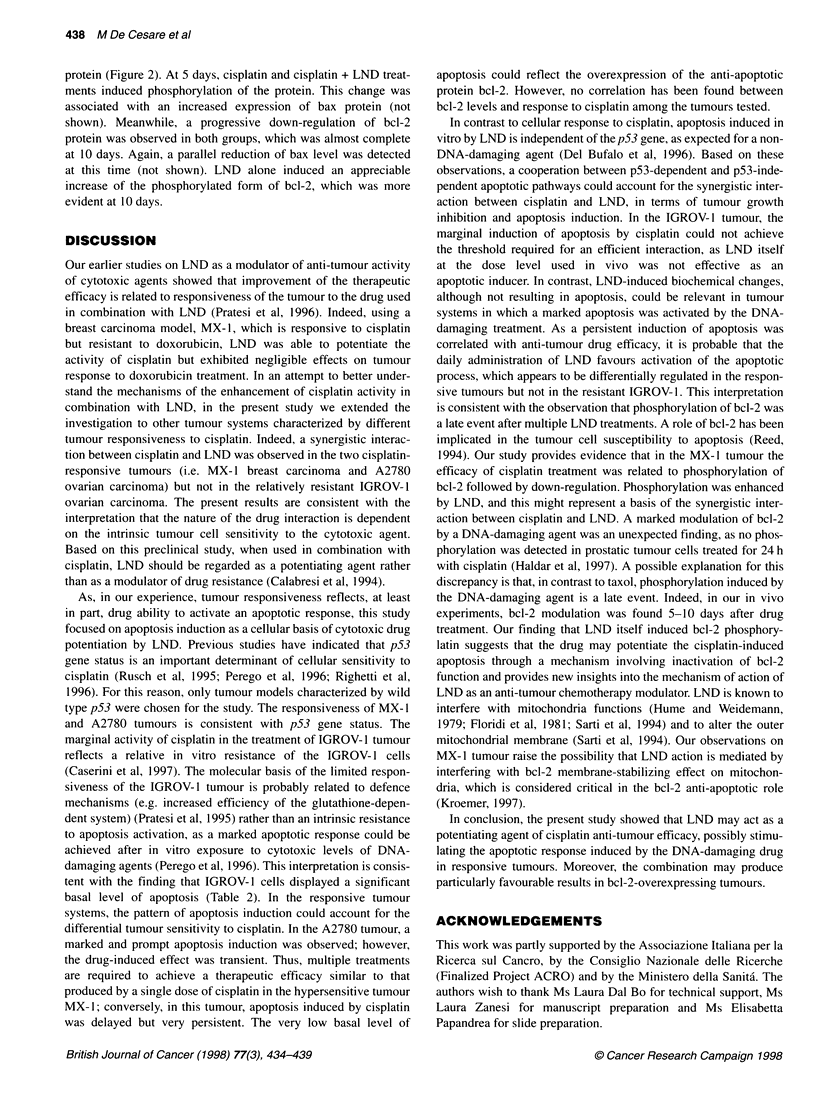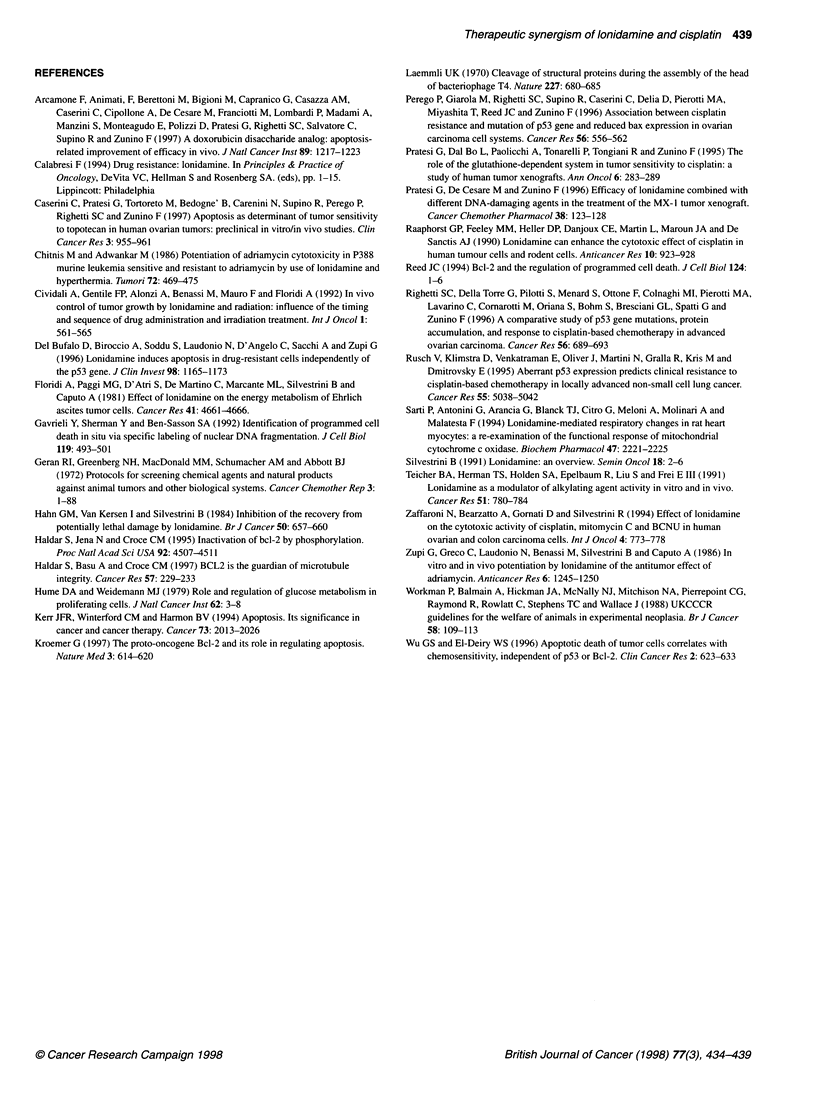# Stimulation of the apoptotic response as a basis for the therapeutic synergism of lonidamine and cisplatin in combination in human tumour xenografts.

**DOI:** 10.1038/bjc.1998.69

**Published:** 1998

**Authors:** M. De Cesare, G. Pratesi, A. Giusti, D. Polizzi, F. Zunino

**Affiliations:** Division of Experimental Oncology B, Istituto Nazionale per lo Studio e la Cura dei Tumori, Milan, Italy.

## Abstract

**Images:**


British Journal of Cancer (1998) 77(3), 434–439
© 1998 Cancer Research Campaign

### Stimulation of the apoptotic response as a basis for the therapeutic synergism of lonidamine and cisplatin in combination in human tumour xenografts

**M De Cesare[1], G Pratesi[1], A Giusti[2], D Polizzi[1] and F Zunino[1]**

[1]Division of Experimental Oncology B, Istituto Nazionale per lo Studio e la Cura dei Tumori, Via Venezian 1, 20133 Milan; [2]Istituto di Anatomia Patologica Veterinaria e Patologia Aviarie, via Celoria 10, 20133 Milan, Italy

**Summary** The pharmacological interest in lonidamine is related to its ability to enhance the cytotoxic effects of several DNA-damaging anti-tumour agents. This study was undertaken to better understand the in vivo interaction between lonidamine and cisplatin in the treatment of human tumour xenografts, including three carcinoma models characterized by a different responsiveness to cisplatin, in spite of the presence of a wild-type *p53* gene in all tumours. The drug combination was more effective in tumour growth inhibition than cisplatin alone against MX-1 breast carcinoma and A2780 ovarian carcinoma, both highly responsive to cisplatin, whereas no influence of lonidamine was observed on anti-tumour activity of cisplatin in the treatment of the relatively resistant IGROV-1 ovarian carcinoma. As cisplatin activity is related to induction of apoptosis, the modulation of drug-induced apoptosis by lonidamine was investigated. Under conditions in which lonidamine itself had negligible effects on tumour growth and apoptosis, the modulating agent stimulated the apoptotic response induced by cisplatin in the responsive but not in the resistant tumours. Tumour response was dependent not only on the drug activation of apoptosis, but mainly on the persistence over time of the event. In the breast carcinoma MX-1, hypersensitive to cisplatin and to the lonidamine+cisplatin combination, the efficacy of drug treatment was associated with phosphorylation of bcl-2 followed by down-regulation of the protein. Lonidamine itself caused a delayed phosphorylation of bcl-2. These results are consistent with the interpretation that lonidamine is effective in modulating biochemical factors involved in regulation of apoptosis.

**Keywords:** lonidamine; cisplatin; apoptosis; bcl-2; p53

Lonidamine (LND) is a dichlorinated derivative of indazole-3-carboxylic acid, which has been described as an inhibitor of energy metabolism (Silvestrini, 1991). A selective action of LND on tumour cells has been attributed to its inhibitory effect on mitochondria-bound hexokinase, which is present in tumour cells but not in normal differentiated cells (Hume and Weidemann, 1979; Floridi et al, 1981). Studies on different tumour cell lines have shown increased cytotoxic efficacy of DNA-damaging anti-tumour drugs when combined to LND (Chitnis and Adwankar, 1986; Zupi et al, 1986; Raaphorst et al, 1990; Teicher et al, 1991; Zaffaroni et al, 1994). In the human MX-1 mammary tumour xenografted in nude mice, we recently demonstrated a therapeutic advantage for LND combined to cisplatin (Pratesi et al, 1996). The cellular and molecular basis of the interaction remains to be determined. At the cellular level, the synergism could be related to a specific interference of LND with energy-dependent DNA-repair processes subsequent to cisplatin-induced DNA damage (Hahn et al, 1984). Such an effect could result in a fixation of DNA damage, thus activating a cascade of biochemical events, possibly resulting in cell death by apoptosis. Indeed, apoptosis is a major mode of cell death in response to DNA-damaging treatment (Kerr et al, 1994), and a correlation between apoptotic death and tumour sensitivity to drugs has been documented in some in vitro systems (Wu and El-Deiry, 1996). We recently reported that human ovarian cancer cell lines selected for resistance to cisplatin have reduced susceptibility to apoptosis induction after cytotoxic treatment, as a consequence of loss of wild-type *p53* function (Perego et al, 1996). A correlation between apoptosis and cellular sensitivity has also been documented for LND, but LND-induced apoptosis is not mediated by the *p53* gene (Del Bufalo et al, 1996).

The aim of the present study was to investigate further the interaction of LND and cisplatin in human carcinoma systems characterized by different responsiveness to cisplatin. One relatively resistant (the ovarian IGROV-1) and two sensitive (the ovarian A2780 and the mammary MX-1) carcinomas were used. All tumours, maintained by serial passages in nude female athymic mice, were characterized by wild-type *p53* (Perego et al, 1996; Arcamone et al, 1997; Caserini et al, 1997). In particular, the study investigated the effect of drug treatment on tumour apoptosis induction and its correlation to tumour response. The results of our study showed that LND, under conditions in which alone it had a negligible effect, potentiated cisplatin efficacy only in tumours highly responsive to cisplatin and that, in these sensitive tumour systems, LND enhanced the apoptotic response induced by treatment with the cytotoxic agent and maintained it for a long time. Moreover, our results show that both cisplatin and LND induce phosphorylation of bcl-2 protein.

*Received 19 March 1997*
*Revised 3 July 1997*
*Accepted 8 July 1997*

*Correspondence to:* G Pratesi, Istituto Nazionale Tumori, Via Venezian 1, 20133 Milan, Italy

**Table 1** Influence of LND on anti-tumour activity of cisplatin in human tumour xenografts[a]

| Tumour | Drug | Dose (mg $kg^{-1}$) | Treatment | | TVI%[b] | LCK[c] | Tox/Tot[d] |
|---|---|---|---|---|---|---|---|
| | | | Route | Schedule | | | |
| MX-1 | LND | 50 | i.p. | qd×20 | 33 (39) | 0.2 | 0/5 |
| | Cisplatin | 6 | i.p. | Single treatment | 99 (33) | 3.4 | 0/5 |
| | Cisplatin + LND | 6 + 50 | | | 100[e] (39) | > 7.3 | 0/5 |
| A2780 | LND | 50 | i.p. | qd×20 | 51 (20) | 0.5 | 3/5 |
| | Cisplatin | 6 | i.p. | Single treatment | 65 (17) | 0.5 | 0/5 |
| | Cisplatin + LND | 6 + 50 | | | 78* (20) | 1.3 | 2/5 |
| | LND | 100 | p.o. | qd×5/w×3w | 10 (26) | 0 | 0/5 |
| | Cisplatin | 6 | i.v. | q7d×3 | 90 (26) | 2.0 | 0/5 |
| | Cisplatin+LND | 6 + 100 | | | 97* (26) | 2.8 | 1/5 |
| IGROV-1 | LND | 50 | i.p. | qd×20 | 17 (28) | 0 | 1/5 |
| | Cisplatin | 6 | i.p. | Single treatment | 32 (21) | 0.1 | 0/5 |
| | Cisplatin+LND | 6 + 50 | | | 45 (28) | 0.3 | 1/5 |
| | LND | 100 | p.o. | qd×5/w×3w | 45 (30) | 0.9 | 0/4 |
| | Cisplatin | 6 | i.v. | q7d×3 | 56 (30) | 1.1 | 1/5 |
| | Cisplatin+LND | 6 + 100 | | | 56 (30) | 1.0 | 0/5 |

[a]LND treatment started immediately after each cisplatin injection. [b]The highest inhibition of tumour volume is reported; in parentheses the day on which it was evaluated. [c]$Log_{10}$ cell kill calculated by assessing the difference in mean time required for tumours of treated and control mice to reach 1000 or 2000 $mm^3$ and dividing that value by the product of 3.32 and tumour doubling time. [d]Toxic deaths/total no. of mice. [e]All mice were cured in this group. *$P < 0.05$ vs cisplatin-treated tumours.

### MATERIALS AND METHODS

### Animals

Six- to 10-week-old female athymic nude CD-1 mice were used in the study. Animals (obtained from Charles River, Calco, Italy) were maintained in laminar air-flow rooms. Sterilized cages, bedding and acidified water were used for mice care. The air was conditioned at a temperature of 24–26°C and 50% humidity. The experiments were approved by the Ethics Committee of our Institute, according to UKCCCR guidelines (Workman et al, 1988).

### Tumours

The MX-1 human breast tumour line, from an infiltrating duct cell breast carcinoma of an untreated patient, was established s.c. in nude mice in the National Cancer Institute (NCI, Bethesda, MD, USA). A2780 and IGROV-1 cell lines were derived from ovarian carcinomas of untreated patients. The A2780 and IGROV-1 human tumour lines were established s.c. in nude mice, from the respective cell lines, in the Istituto Nazionale per lo Studio e la Cura dei Tumori, Milan. For in vivo line maintenance and experimental purposes, tumour specimens were grafted s.c. in both flanks of mice by a 13-gauge trocar. Growth of s.c. tumours was followed by biweekly calliper measurements of length and width. Tumour volume (TV) was calculated using the formula: TV = $mm^3$ = width × width × length/2 (Geran et al, 1972). Tumour doubling time was calculated for each tumour line from the semi-logarithmic best-fit curve of each control tumour plotted vs time, from the day on which the tumour became measurable until the day on which the curve began to level off (exponential phase of growth). The pattern of lactic dehydrogenase human isoenzymes could be detected persistently in tumour extracts.

### Chemotherapy studies

Cisplatin (kindly supplied by Pharmacia Upjohn, Milan, Italy) was dissolved in saline and LND (kindly supplied by Angelini, Rome, Italy) was dissolved in 2.3% *N*-methyl-D-glucamine (NMG, Aldrich) water solution; 10 mg of LND was previously dissolved in 0.3 ml of the NMG solution and then diluted in sterile distilled water. Both drugs were given in a volume of 10 mg $kg^{-1}$ of body weight. Each experimental group consisted of at least eight assessable tumours. Control mice were treated with the NMG solution according to the same route and schedule used for LND in the same experiment. Tumour-bearing mice were treated according to two schedules: (a) single i.p. cisplatin treatment followed by 20 daily LND treatments (50 mg $kg^{-1}$) according to the same route and (b) three i.v. cisplatin injections, once a week for 3 consecutive weeks, each one followed by five administrations of LND per os (100 mg $kg^{-1}$). The same dose of cisplatin, 6 mg $kg^{-1}$ per injection, was used in the two schedules. A LND dose of 50 mg $kg^{-1}$ i.p. and 100 mg $kg^{-1}$ per os had similar efficacy in our previous study (Pratesi et al, 1996). In both schedules, LND was delivered after cisplatin treatment because this drug sequence was found to be the optimal one for the combination (Cividali et al, 1992).

Drug activity was assessed as: (a) the percentage of tumour volume inhibition (TVI%), calculated by the formula: TVI% = 100–(tV/cV×100), where tV is the mean TV of treated tumours and cV that of control tumours (the day on which TVI% was calculated is reported in Table 1); (b) the $\log_{10}$ cell kill achieved by the drug treatment, according to the formula: T – C/DT × 3.32, where T is the mean time (days) required for the treated tumours and C the mean time for the control tumours to reach an established volume (see Table 1) and DT is the doubling time of control tumours. A tumour was considered responsive when the drug treatment achieved a $\log_{10}$ cell kill ≥ 1.

© Cancer Research Campaign 1998

**Figure 1** Time course of apoptosis induction in human MX-1, A2780 and IGROV-1 tumour xenografts, treated with: 10 mg kg$^{-1}$ of cisplatin (□, i.p. single injection at day 0); 100 mg kg$^{-1}$ of LND (○, p.o. daily from day 0); or cisplatin + LND (●). Δ, Untreated controls. Mice were killed at the indicated times, tumours were removed immediately and processed as indicated in Materials and methods. Determination of the apoptotic index was assessed at the selected time points. For each time point two tumours were examined. Using a light microscope at ×400 magnification, the mean number of cells per field for each tumour model was assessed examining ten randomly chosen fields (277, 358 and 379 cells per field for MX-1, A2780 and IGROV-1 respectively). Ten fields of non-necrotic areas were selected in each section, in each field the number of apoptotic nuclei was recorded, and the apoptotic index is expressed as percentage of apoptotic nuclei relative to the mean number of cells per field. Mean values are reported. Standard error never exceeded 0.65

Deaths occurring in treated mice before the death of the first control mouse were ascribed to toxic effects.

### Determination of mitotic and apoptotic indexes

Tumour-bearing mice were treated with cisplatin and LND, alone or in combination. Cisplatin was given as a single i.p. injection at the dose of 10 mg kg$^{-1}$ (which is slightly below the maximum-tolerated dose of 12 mg kg$^{-1}$ in our strain of mice); LND (100 mg kg$^{-1}$) was administered orally and daily, starting just after the cisplatin injection. At sequential times after the cisplatin injection (from 1 day on), one mouse per group was sacrificed by cervical dislocation; its two tumours were removed, placed in neutral-buffered formalin and processed for embedding in paraffin blocks. From each tumour block, 2- to 4-micron sections were cut and adhered to poly-L-lysine pretreated slides. Tissue sections were then treated according to the method described by Gavrieli et al (1992). For the TdT-mediated dUTP nick-end labelling (TUNEL) reaction, the in situ cell death detection POD kit (Boehringer Mannheim, Ingelheim, Germany) was used. The final staining was performed using a DAB (Sigma, St Louis, MO, USA) solution. Sections were counterstained with haematoxylin. Apoptotic index (AI) was determined as indicated in the figure legends, using a light microscope at 400× magnification. Successive sections of the same tumour samples were stained with the standard haematoxylin–eosin method to determine the mitotic index (MI), which is expressed as for the AI. Scoring of histological tumour sections was conducted without knowledge of the results of the tumour growth-inhibition studies.

### Western blot analysis

Tumour samples were minced and suspended in 500 µl of Laemmli buffer (Laemmli, 1970); suspensions were completely disrupted using a tissue Potter and successively sonicated for 1 min at low intensity. Debris was sedimented at 12 000 r.p.m. for 10 min at 4°C. Supernatants containing protein extracts were collected, and a 1:10 volume of β-mercaptoethanol was added; they were then boiled for 3 min and stored at –80°C. Protein concentration was determined using the BCA Protein Assay Reagent (Pierce, Rockford, IL USA). Aliquots of 80 µg were size-separated by SDS-PAGE (12% gel) and transferred to a nitrocellulose filter (BioRad Lab, Segrate, Italy) by electroblotting. Immunodetection was carried out using an anti-bcl-2 (Santa Cruz Biotechnology, Segrate, Italy) polyclonal antibody (1:1000), and blots were successively developed with peroxidase anti-rabbit antibody (1:2000) using the enhanced chemiluminescence (ECL) detection kit (Amersham, Little Chalfont, UK). The phosphorylated form of bcl-2 protein was detected as a slower mobility form as previously described (Haldar et al, 1995).

### Statistical analysis

The statistical significance of the differences in tumour volumes and AI values between cisplatin- and cisplatin + LND-treated tumours was tested using the Student's *t*-test (two-tailed). *P*-values were considered to be significant when less than or equal to 0.05.

### RESULTS

### Chemotherapy studies

The influence of LND on cisplatin anti-tumour efficacy in human tumour xenografts is reported in Table 1. The MX-1 tumour was very sensitive to cisplatin treatment, as tumours treated with a single suboptimal dose disappeared by day 15. However, some tumours regrew after 40–50 days (Pratesi et al, 1996). The enhancement induced by LND on this tumour was dramatic, as all mice were cured by the combination (no tumour regrowth up to 100 days). A significant enhancement of cisplatin anti-tumour activity by LND was achieved also in A2780 tumour-bearing mice, using either treatment schedules. As expected, in this sensitive tumour,

© Cancer Research Campaign 1998

**Table 2** Effect of cisplatin and the cisplatin + lonidamine combination on apoptosis in human carcinoma xenografts

| Tumour line (type) | DT[a] (days) | Mitosis Basal[b] | Apoptosis Basal[b] | Apoptosis Peak[c] (day) Cisplatin | Apoptosis Peak[c] (day) Cisplatin + LND |
|---|---|---|---|---|---|
| MX-1 (mammary) | 6.3 ± 0.9 | 3.4 ± 0.6 | 0.4 ± 0.2 | 4.35 (10) | 6 (10) |
| A2780 (ovarian) | 2.4 ± 0.5 | 0.8 ± 0.18 | 1.4 ± 0.2 | 10.95 (1) | 18.1 (6) |
| IGROV-1 (ovarian) | 4.9 ± 1.1 | 0.7 ± 0.2 | 1.8 ± 0.5 | 3.7 (1) | 3.4 (1) |

[a]Doubling time (DT) was determined in untreated control tumours. Mean values ± s.d. are shown. [b]The basal level of mitosis and apoptosis was determined from all untreated control tumours. Mean values ± s.d. are shown. [c]The peak level of apoptosis was determined from the time course of treated tumours and is defined as the highest AI value observed within the observation time. In parentheses, the day at which the peak was detected.

**Figure 2** Western blot analysis of bcl-2 protein in human MX-1 tumour. Tumour-bearing mice were treated with 10 mg kg$^{-1}$ of cisplatin (day 0, i.v.; lane 2), cisplatin + LND (lane 3), 100 mg kg$^{-1}$ of LND (p.o. daily from day 0; lane 4) or were untreated (lane 1). Five (**A**) and 10 days (**B**) after cisplatin treatment, tumours were excised and processed as described in Materials and methods. Equal amounts of protein were loaded. Arrows, modified (phosphorylated) form of bcl-2 protein cisplatin activity was higher when the optimal dose (6 mg kg$^{-1}$ for three times) instead of a suboptimal one (6 mg kg$^{-1}$, single treatment) was administered to mice. Conversely, only a marginal increase of drug efficacy was observed in the naturally resistant IGROV-1 tumour between one and three cisplatin treatments, nor did LND influence the low anti-tumour activity. LND was better tolerated after oral administration than i.p. treatment.

### Induction of apoptosis

The kinetics of apoptosis induction by drug treatment was studied in tumour tissues, and the time course and the extent of apoptosis induction in the three tumour lines is reported in Figure 1. Established MX-1 tumours (450-mm$^3$ mean volume) were treated with 10 mg kg$^{-1}$ of cisplatin, and the AI was calculated in histological sections of tumours at sequential times (from day 1 to day 12 after treatment). In cisplatin-treated tumours, the percentage of apoptotic cells was similar to that of untreated controls on day 1 and rose to high values from day 5 onwards. The AI in tumours treated with cisplatin + LND (given daily) followed the same kinetics as that of cisplatin-treated tumours but was generally higher (most evident on day 10). The increase in AI of treated tumours was persistent, as the AI was still higher than that of control tumours on the last day of observation (day 12). No evaluation could be made at longer times for tumour disappearance. Untreated control tumours presented a low AI (basal level 0.4 ± 0.2), which remained unchanged up to day 12. LND alone did not modify the AI compared with untreated controls.

Mice bearing A2780 tumours were treated when tumour volume was around 350 mm$^3$. Spontaneous tumour apoptosis was higher (basal level 1.4 ± 0.2) than that in MX-1 tumours. Again, LND alone was ineffective as an apoptosis inducer at all times. In cisplatin-treated tumours, a prompt increase in AI had already been induced at 1 day after treatment, but values returned to basal levels by day 6. Conversely, in the cisplatin + LND-treated tumours the high level of AI lasted much longer, and the level at day 6 was significantly different between the two groups ($P < 0.001$).

In IGROV-1 tumour-bearing mice, treated when tumours were about 200 mm$^3$, basal apoptosis values were the highest among the three tumours tested (basal level 1.8 ± 0.5). In spite of tumour cell ability to activate spontaneous apoptosis, cisplatin treatment only marginally increased the AI (less than twofold) and only for a short time (peak level on day 1). Moreover, the AI in tumours treated with the cisplatin + LND combination never exceeded the values of cisplatin-treated tumours.

Table 2 summarizes the apoptotic and mitotic levels in the three tumour lines investigated in the study. Mitosis level differed in the tumours and did not correlate with tumour doubling time. The MX-1 tumour, which has the highest MI, was also the most responsive tumour to cisplatin treatment. Considering the apoptosis level induced by cisplatin treatment, it is noteworthy that the very sensitive MX-1 tumour cells keep dying from apoptosis for long time (the AI was still higher than in control tumours after 12 days). Moreover, the duration of apoptosis correlated well with tumour response to treatment. Comparing the time course of apoptosis in MX-1 and A2780 tumours, the influence of LND on apoptosis induced by cisplatin appeared to be related more to maintenance than to the peak level of the apoptotic event.

### Bcl-2 expression and modulation

Although all three tumours were characterized by a wild type *p53*, only MX-1 overexpressed bcl-2 protein. Thus, only in this tumour model, modulation of the anti-apoptic protein was studied after treatment with cisplatin (single i.v. 10 mg kg$^{-1}$) and/or LND (daily per os, 100 mg kg$^{-1}$). Five and 10 days after cisplatin treatment (day 0), one mouse per group was sacrificed and its two tumours were excised and processed for Western blot analysis of bcl-2

© Cancer Research Campaign 1998

protein (Figure 2). At 5 days, cisplatin and cisplatin + LND treatments induced phosphorylation of the protein. This change was associated with an increased expression of bax protein (not shown). Meanwhile, a progressive down-regulation of bcl-2 protein was observed in both groups, which was almost complete at 10 days. Again, a parallel reduction of bax level was detected at this time (not shown). LND alone induced an appreciable increase of the phosphorylated form of bcl-2, which was more evident at 10 days.

### DISCUSSION

Our earlier studies on LND as a modulator of anti-tumour activity of cytotoxic agents showed that improvement of the therapeutic efficacy is related to responsiveness of the tumour to the drug used in combination with LND (Pratesi et al, 1996). Indeed, using a breast carcinoma model, MX-1, which is responsive to cisplatin but resistant to doxorubicin, LND was able to potentiate the activity of cisplatin but exhibited negligible effects on tumour response to doxorubicin treatment. In an attempt to better understand the mechanisms of the enhancement of cisplatin activity in combination with LND, in the present study we extended the investigation to other tumour systems characterized by different tumour responsiveness to cisplatin. Indeed, a synergistic interaction between cisplatin and LND was observed in the two cisplatin-responsive tumours (i.e. MX-1 breast carcinoma and A2780 ovarian carcinoma) but not in the relatively resistant IGROV-1 ovarian carcinoma. The present results are consistent with the interpretation that the nature of the drug interaction is dependent on the intrinsic tumour cell sensitivity to the cytotoxic agent. Based on this preclinical study, when used in combination with cisplatin, LND should be regarded as a potentiating agent rather than as a modulator of drug resistance (Calabresi et al, 1994).

As, in our experience, tumour responsiveness reflects, at least in part, drug ability to activate an apoptotic response, this study focused on apoptosis induction as a cellular basis of cytotoxic drug potentiation by LND. Previous studies have indicated that *p53* gene status is an important determinant of cellular sensitivity to cisplatin (Rusch et al, 1995; Perego et al, 1996; Righetti et al, 1996). For this reason, only tumour models characterized by wild type *p53* were chosen for the study. The responsiveness of MX-1 and A2780 tumours is consistent with *p53* gene status. The marginal activity of cisplatin in the treatment of IGROV-1 tumour reflects a relative in vitro resistance of the IGROV-1 cells (Caserini et al, 1997). The molecular basis of the limited responsiveness of the IGROV-1 tumour is probably related to defence mechanisms (e.g. increased efficiency of the glutathione-dependent system) (Pratesi et al, 1995) rather than an intrinsic resistance to apoptosis activation, as a marked apoptotic response could be achieved after in vitro exposure to cytotoxic levels of DNA-damaging agents (Perego et al, 1996). This interpretation is consistent with the finding that IGROV-1 cells displayed a significant basal level of apoptosis (Table 2). In the responsive tumour systems, the pattern of apoptosis induction could account for the differential tumour sensitivity to cisplatin. In the A2780 tumour, a marked and prompt apoptosis induction was observed; however, the drug-induced effect was transient. Thus, multiple treatments are required to achieve a therapeutic efficacy similar to that produced by a single dose of cisplatin in the hypersensitive tumour MX-1; conversely, in this tumour, apoptosis induced by cisplatin was delayed but very persistent. The very low basal level of apoptosis could reflect the overexpression of the anti-apoptotic protein bcl-2. However, no correlation has been found between bcl-2 levels and response to cisplatin among the tumours tested.

In contrast to cellular response to cisplatin, apoptosis induced in vitro by LND is independent of the *p53* gene, as expected for a non-DNA-damaging agent (Del Bufalo et al, 1996). Based on these observations, a cooperation between p53-dependent and p53-independent apoptotic pathways could account for the synergistic interaction between cisplatin and LND, in terms of tumour growth inhibition and apoptosis induction. In the IGROV-1 tumour, the marginal induction of apoptosis by cisplatin could not achieve the threshold required for an efficient interaction, as LND itself at the dose level used in vivo was not effective as an apoptotic inducer. In contrast, LND-induced biochemical changes, although not resulting in apoptosis, could be relevant in tumour systems in which a marked apoptosis was activated by the DNA-damaging treatment. As a persistent induction of apoptosis was correlated with anti-tumour drug efficacy, it is probable that the daily administration of LND favours activation of the apoptotic process, which appears to be differentially regulated in the responsive tumours but not in the resistant IGROV-1. This interpretation is consistent with the observation that phosphorylation of bcl-2 was a late event after multiple LND treatments. A role of bcl-2 has been implicated in the tumour cell susceptibility to apoptosis (Reed, 1994). Our study provides evidence that in the MX-1 tumour the efficacy of cisplatin treatment was related to phosphorylation of bcl-2 followed by down-regulation. Phosphorylation was enhanced by LND, and this might represent a basis of the synergistic interaction between cisplatin and LND. A marked modulation of bcl-2 by a DNA-damaging agent was an unexpected finding, as no phosphorylation was detected in prostatic tumour cells treated for 24 h with cisplatin (Haldar et al, 1997). A possible explanation for this discrepancy is that, in contrast to taxol, phosphorylation induced by the DNA-damaging agent is a late event. Indeed, in our in vivo experiments, bcl-2 modulation was found 5–10 days after drug treatment. Our finding that LND itself induced bcl-2 phosphorylatin suggests that the drug may potentiate the cisplatin-induced apoptosis through a mechanism involving inactivation of bcl-2 function and provides new insights into the mechanism of action of LND as an anti-tumour chemotherapy modulator. LND is known to interfere with mitochondria functions (Hume and Weidemann, 1979; Floridi et al, 1981; Sarti et al, 1994) and to alter the outer mitochondrial membrane (Sarti et al, 1994). Our observations on MX-1 tumour raise the possibility that LND action is mediated by interfering with bcl-2 membrane-stabilizing effect on mitochondria, which is considered critical in the bcl-2 anti-apoptotic role (Kroemer, 1997).

In conclusion, the present study showed that LND may act as a potentiating agent of cisplatin anti-tumour efficacy, possibly stimulating the apoptotic response induced by the DNA-damaging drug in responsive tumours. Moreover, the combination may produce particularly favourable results in bcl-2-overexpressing tumours.

### ACKNOWLEDGEMENTS

This work was partly supported by the Associazione Italiana per la Ricerca sul Cancro, by the Consiglio Nazionale delle Ricerche (Finalized Project ACRO) and by the Ministero della Sanitá. The authors wish to thank Ms Laura Dal Bo for technical support, Ms Laura Zanesi for manuscript preparation and Ms Elisabetta Papandrea for slide preparation.

© Cancer Research Campaign 1998

### REFERENCES

Arcamone F, Animati, F, Berettoni M, Bigioni M, Capranico G, Casazza AM, Caserini C, Cipollone A, De Cesare M, Franciotti M, Lombardi P, Madami A, Manzini S, Monteagudo E, Polizzi D, Pratesi G, Righetti SC, Salvatore C, Supino R and Zunino F (1997) A doxorubicin disaccharide analog: apoptosis-related improvement of efficacy in vivo. *J Natl Cancer Inst* **89**: 1217–1223

Calabresi F (1994) Drug resistance: lonidamine. In *Principles & Practice of Oncology*, DeVita VC, Hellman S and Rosenberg SA. (eds), pp. 1–15. Lippincott: Philadelphia

Caserini C, Pratesi G, Tortoreto M, Bedogne' B, Carenini N, Supino R, Perego P, Righetti SC and Zunino F (1997) Apoptosis as determinant of tumor sensitivity to topotecan in human ovarian tumors: preclinical in vitro/in vivo studies. *Clin Cancer Res* **3**: 955–961

Chitnis M and Adwankar M (1986) Potentiation of adriamycin cytotoxicity in P388 murine leukemia sensitive and resistant to adriamycin by use of lonidamine and hyperthermia. *Tumori* **72**: 469–475

Cividali A, Gentile FP, Alonzi A, Benassi M, Mauro F and Floridi A (1992) In vivo control of tumor growth by lonidamine and radiation: influence of the timing and sequence of drug administration and irradiation treatment. *Int J Oncol* **1**: 561–565

Del Bufalo D, Biroccio A, Soddu S, Laudonio N, D'Angelo C, Sacchi A and Zupi G (1996) Lonidamine induces apoptosis in drug-resistant cells independently of the p53 gene. *J Clin Invest* **98**: 1165–1173

Floridi A, Paggi MG, D'Atri S, De Martino C, Marcante ML, Silvestrini B and Caputo A (1981) Effect of lonidamine on the energy metabolism of Ehrlich ascites tumor cells. *Cancer Res* **41**: 4661–4666.

Gavrieli Y, Sherman Y and Ben-Sasson SA (1992) Identification of programmed cell death in situ via specific labeling of nuclear DNA fragmentation. *J Cell Biol* **119**: 493–501

Geran RI, Greenberg NH, MacDonald MM, Schumacher AM and Abbott BJ (1972) Protocols for screening chemical agents and natural products against animal tumors and other biological systems. *Cancer Chemother Rep* **3**: 1–88

Hahn GM, Van Kersen I and Silvestrini B (1984) Inhibition of the recovery from potentially lethal damage by lonidamine. *Br J Cancer* **50**: 657–660

Haldar S, Jena N and Croce CM (1995) Inactivation of bcl-2 by phosphorylation. *Proc Natl Acad Sci USA* **92**: 4507–4511

Haldar S, Basu A and Croce CM (1997) BCL2 is the guardian of microtubule integrity. *Cancer Res* **57**: 229–233

Hume DA and Weidemann MJ (1979) Role and regulation of glucose metabolism in proliferating cells. *J Natl Cancer Inst* **62**: 3–8

Kerr JFR, Winterford CM and Harmon BV (1994) Apoptosis. Its significance in cancer and cancer therapy. *Cancer* **73**: 2013–2026

Kroemer G (1997) The proto-oncogene Bcl-2 and its role in regulating apoptosis. *Nature Med* **3**: 614–620

Laemmli UK (1970) Cleavage of structural proteins during the assembly of the head of bacteriophage T4. *Nature* **227**: 680–685

Perego P, Giarola M, Righetti SC, Supino R, Caserini C, Delia D, Pierotti MA, Miyashita T, Reed JC and Zunino F (1996) Association between cisplatin resistance and mutation of p53 gene and reduced bax expression in ovarian carcinoma cell systems. *Cancer Res* **56**: 556–562

Pratesi G, Dal Bo L, Paolicchi A, Tonarelli P, Tongiani R and Zunino F (1995) The role of the glutathione-dependent system in tumor sensitivity to cisplatin: a study of human tumor xenografts. *Ann Oncol* **6**: 283–289

Pratesi G, De Cesare M and Zunino F (1996) Efficacy of lonidamine combined with different DNA-damaging agents in the treatment of the MX-1 tumor xenograft. *Cancer Chemother Pharmacol* **38**: 123–128

Raaphorst GP, Feeley MM, Heller DP, Danjoux CE, Martin L, Maroun JA and De Sanctis AJ (1990) Lonidamine can enhance the cytotoxic effect of cisplatin in human tumour cells and rodent cells. *Anticancer Res* **10**: 923–928

Reed JC (1994) Bcl-2 and the regulation of programmed cell death. *J Cell Biol* **124**: 1–6

Righetti SC, Della Torre G, Pilotti S, Menard S, Ottone F, Colnaghi MI, Pierotti MA, Lavarino C, Cornarotti M, Oriana S, Bohm S, Bresciani GL, Spatti G and Zunino F (1996) A comparative study of p53 gene mutations, protein accumulation, and response to cisplatin-based chemotherapy in advanced ovarian carcinoma. *Cancer Res* **56**: 689–693

Rusch V, Klimstra D, Venkatraman E, Oliver J, Martini N, Gralla R, Kris M and Dmitrovsky E (1995) Aberrant p53 expression predicts clinical resistance to cisplatin-based chemotherapy in locally advanced non-small cell lung cancer. *Cancer Res* **55**: 5038–5042

Sarti P, Antonini G, Arancia G, Blanck TJ, Citro G, Meloni A, Molinari A and Malatesta F (1994) Lonidamine-mediated respiratory changes in rat heart myocytes: a re-examination of the functional response of mitochondrial cytochrome c oxidase. *Biochem Pharmacol* **47**: 2221–2225

Silvestrini B (1991) Lonidamine: an overview. *Semin Oncol* **18**: 2–6

Teicher BA, Herman TS, Holden SA, Epelbaum R, Liu S and Frei E III (1991) Lonidamine as a modulator of alkylating agent activity in vitro and in vivo. *Cancer Res* **51**: 780–784

Zaffaroni N, Bearzatto A, Gornati D and Silvestrini R (1994) Effect of lonidamine on the cytotoxic activity of cisplatin, mitomycin C and BCNU in human ovarian and colon carcinoma cells. *Int J Oncol* **4**: 773–778

Zupi G, Greco C, Laudonio N, Benassi M, Silvestrini B and Caputo A (1986) In vitro and in vivo potentiation by lonidamine of the antitumor effect of adriamycin. *Anticancer Res* **6**: 1245–1250

Workman P, Balmain A, Hickman JA, McNally NJ, Mitchison NA, Pierrepoint CG, Raymond R, Rowlatt C, Stephens TC and Wallace J (1988) UKCCCR guidelines for the welfare of animals in experimental neoplasia. *Br J Cancer* **58**: 109–113

Wu GS and El-Deiry WS (1996) Apoptotic death of tumor cells correlates with chemosensitivity, independent of p53 or Bcl-2. *Clin Cancer Res* **2**: 623–633

© Cancer Research Campaign 1998